# Therapeutic Misestimation in Patients with Degenerative Ataxia: Lessons from a Randomized Controlled Trial

**DOI:** 10.1002/mds.29252

**Published:** 2022-10-19

**Authors:** Roderick P.P.W.M. Maas, Bart P.C. van de Warrenburg

**Affiliations:** ^1^ Department of Neurology Donders Institute for Brain, Cognition, and Behaviour, Radboud University Medical Center Nijmegen The Netherlands

**Keywords:** degenerative cerebellar ataxia, placebo effect, expectations of benefit, randomized controlled trial, therapeutic misestimation, therapeutic misconception

## Abstract

**Background:**

The absence of effective treatments may render patients with degenerative cerebellar ataxias susceptible to a placebo response, which could affect the outcome of clinical trials.

**Objective:**

To retrospectively examine expectations of benefit in participants of an ataxia trial and identify determinants of possible therapeutic misestimation.

**Methods:**

Individuals with spinocerebellar ataxia type 3 who participated in a randomized, double‐blind, sham‐controlled trial received a custom‐designed questionnaire about short‐term and long‐term treatment expectations, allocation preferences, and interpretation of treatment arm assignment based on the presence or absence of clinical improvement. To evaluate whether expectations were specifically related to the application of cerebellar transcranial direct current stimulation (tDCS) or more generally reflect an overly positive attitude of patients with ataxia toward trial participation and results, the last questions involved a hypothetical scenario in which an oral drug was tested against placebo with an aim identical to that of our tDCS study.

**Results:**

All 20 trial participants completed the questionnaire. If allocated to the active treatment arm, 75% of patients expected short‐term health benefits and 55% thought they would still have less severe ataxia at 1‐year follow‐up compared with baseline. After 2 weeks, an average reduction in ataxia severity of 31.5% (standard deviation, 22.2%) was anticipated. Conversely, 65% associated a lack of improvement with probable or definite allocation to the placebo group. High expectations of benefit were neither related to the type of intervention nor to clinical or demographic characteristics.

**Conclusion:**

Therapeutic misestimation is common in patients with degenerative ataxia and requires special attention in future trials. © 2022 The Authors. *Movement Disorders* published by Wiley Periodicals LLC on behalf of International Parkinson and Movement Disorder Society.

The degenerative cerebellar ataxias encompass a heterogeneous group of inherited and acquired conditions that share an invariably progressive disease course. Apart from a handful of exceptions, specific interventions that provide clinically meaningful benefits are currently lacking and treatment still focuses on exercise and rehabilitation programs.^1^ Be that as it may, an appreciable number of randomized controlled studies have been conducted during the past 10 years, but mostly with neutral outcomes.^2^

Intriguingly, 3 recent trials from different research groups demonstrated a larger reduction in Scale for the Assessment and Rating of Ataxia (SARA) score, albeit not statistically significant, among patients who received placebo compared with those in the intervention arms.^3^, ^4^, ^5^ Although day‐to‐day fluctuations in ataxia severity and possible practice effects associated with the repeated administration of rating scales might also explain the observed improvement in placebo‐treated individuals, this thought‐provoking finding raises the important question whether and to what extent the placebo effect is implicated in patients with ataxia.

In contrast to other movement disorders, in particular Parkinson's disease, there is a paucity of studies that have directly investigated the placebo response in degenerative ataxias.^6^ Given the lack of effective therapies for these disorders, it is conceivable that patients with ataxia, despite carefully provided written and oral information, have disproportionately high expectations of benefit when invited to eventually participate in a randomized controlled trial. Because patients' expectations are the primary driver of the placebo effect and, as such, could influence the outcome of interventional studies,^7^ we aimed to (1) retrospectively explore these expectations in participants of an ataxia trial and (2) identify demographic and clinical determinants of possible therapeutic misestimation.

## 1 Methods

In May 2021, 20 individuals with spinocerebellar ataxia type 3 (SCA3) who participated in a randomized, double‐blind, sham‐controlled trial of cerebellar transcranial direct current stimulation (tDCS) received a custom‐designed questionnaire about short‐term and long‐term treatment expectations (ie, anticipated changes in ataxia severity after 2 weeks and 1 year of follow‐up, respectively), allocation preferences, and interpretation of treatment arm assignment based on the presence or absence of clinical improvement.^5^ The full questionnaire as well as a timeline of the study can be found in the Supplementary Materials. To evaluate whether expectations of benefit were specifically related to the application of tDCS or more generally reflect an overly positive attitude of patients with ataxia toward trial participation and results, the last questions involved a hypothetical scenario in which an oral drug was tested against placebo with an aim identical to that of our study.

Subjects were eligible for participation in the tDCS trial when they (1) had genetically confirmed SCA3, (2) were aged 16 years or older, and (3) were mildly to moderately affected, as defined by a SARA score between 3 and 20 at a prestudy visit. None of them had previously undergone tDCS. At the time of survey completion, disclosure of treatment assignment had not yet taken place, excluding bias due to knowledge of randomization status. Patients were informed as follows about the possible benefits of participation: “Despite the fact that a recent cerebellar tDCS trial showed a decrease in ataxia severity at the group level in 20 individuals with different types of ataxia that lasted 3 months, it is not known beforehand whether this also applies to SCA3 patients. It is therefore best to assume that there are no specific benefits of participation for yourself. However, if we are able to demonstrate in this study that tDCS reduces ataxia severity, this could have potential consequences for the future treatment of SCA3 patients.”

Outcomes are generally reported using descriptive statistics. An unpaired *t* test was applied to examine if the anticipated change in ataxia severity after 2 weeks of real tDCS differed between patients in both trial arms. Associations between the percentage of expected improvement after 2 weeks of treatment and age, SARA score, disease duration, cerebellar cognitive affective syndrome scale (CCAS‐S) score, educational level, and observed change in SARA score were determined using Pearson correlation coefficients. Finally, a paired‐samples *t* test was applied to analyze possible differences in short‐term expectations of benefit following cerebellar tDCS and a hypothetical oral drug. The level of significance was set at 0.05.

The trial was approved by the local ethics committee (CMO region Arnhem‐Nijmegen). Written informed consent was obtained from all participants.

## 2 Results

All 20 trial participants (12 men; age at baseline, 51.9 ± 10.0 years) completed the questionnaire. SARA score and disease duration at baseline were 11.9 ± 3.9 points and 8.0 ± 5.4 years, respectively.

**FIG 1:**
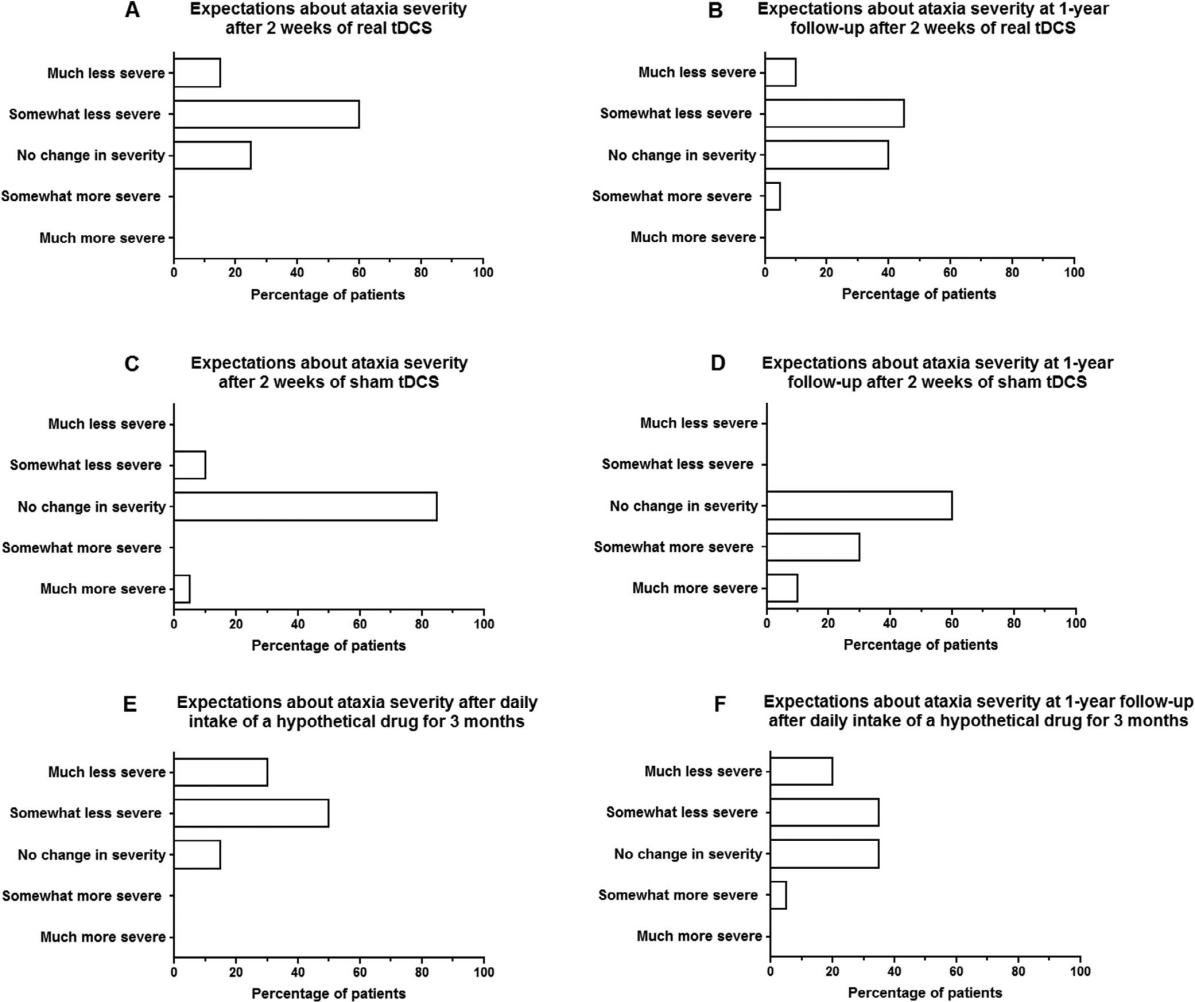
Short‐term and long‐term expectations of benefit of patients with spinocerebellar ataxia type 3 after 10 sessions of real cerebellar transcranial direct current stimulation (tDCS) administered during 2 consecutive weeks (**A**, **B**), after 10 sessions of sham tDCS administered during 2 consecutive weeks (**C**, **D**), and after 3 months of daily intake of a hypothetical oral drug (**E**, **F**). The latter panels reflect a hypothetical scenario in which an oral drug would be tested against placebo in a study with an aim identical to that of our tDCS trial.

**FIG 2:**
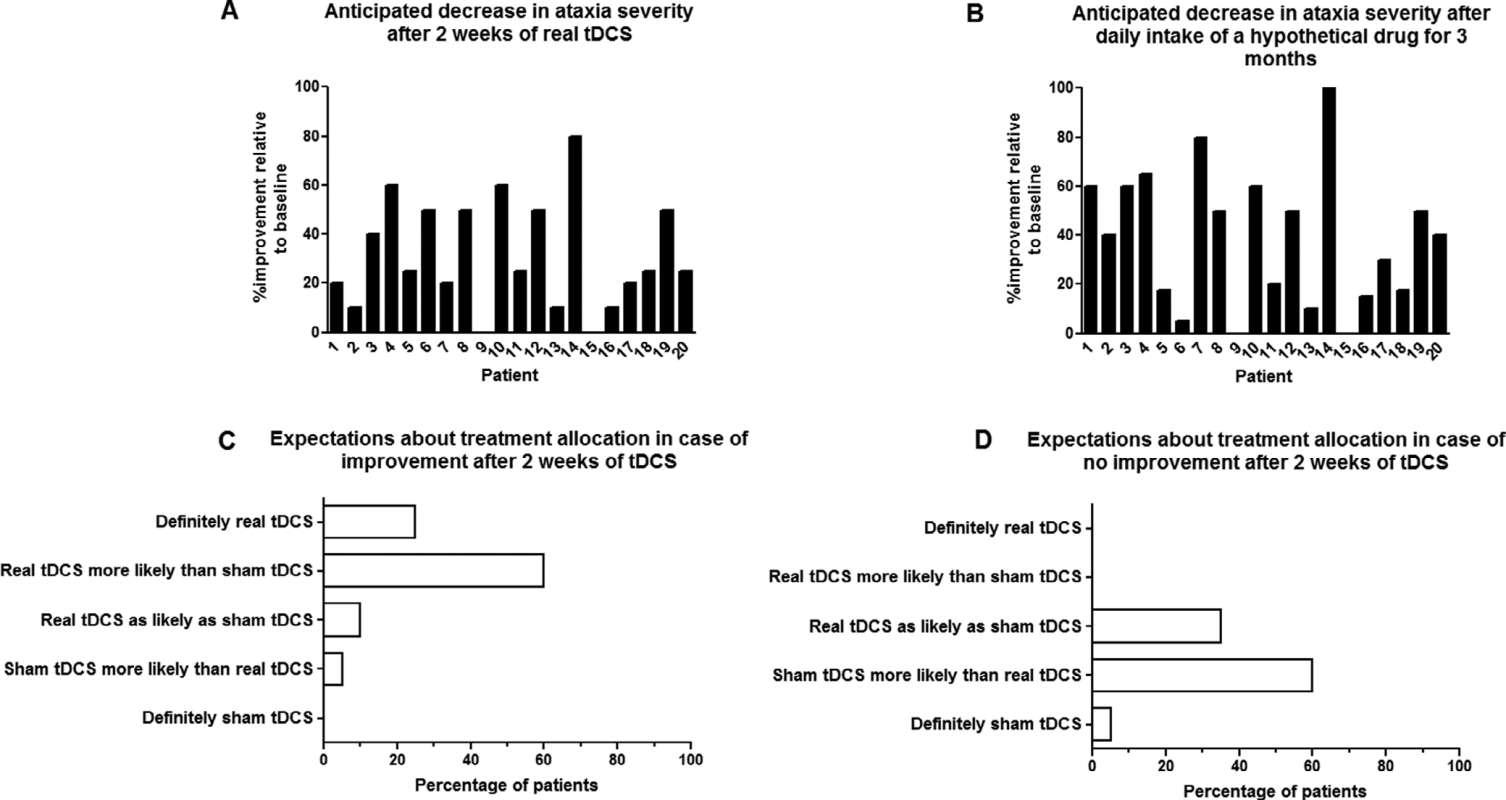
Short‐term expectations of benefit of individual patients with spinocerebellar ataxia type 3 and their interpretation of treatment assignment based on the presence or absence of clinical improvement. The anticipated decrease in ataxia severity is shown after (**A**) 10 sessions of real cerebellar transcranial direct current stimulation (tDCS) and (**B**) after 3 months of daily intake of a hypothetical oral drug. Patients' expectations about treatment allocation after 10 sessions of cerebellar tDCS are shown in the event of (**C**) a decrease in ataxia severity and (**D**) the lack thereof.

Prior to participation, 75% of patients expected to have either somewhat less severe or much less severe ataxic symptoms if they were to receive real tDCS daily for 2 consecutive weeks (Fig. 1A). The remaining quarter believed that their ataxia severity would remain unchanged after these 2 weeks. On average, respondents anticipated to experience a short‐term improvement of 31.5% (standard deviation [SD], 22.2%) following real tDCS (Fig. 2A). In this regard, there was no relevant difference between patients who had been randomly assigned to real tDCS (38.0 ± 25.6%) versus those allocated to sham tDCS (25.0% ± 17.0%) (*P* = 0.20). Only one individual expected a slight worsening at 1‐year follow‐up, while 55% of patients thought that they would still have less severe ataxia compared with baseline (Fig. 1B). When allocated to sham tDCS, no change was expected by 85% of respondents after 2 weeks and by 60% at 1‐year follow‐up (Fig. 1C,D).

In the following questions, patients were asked how they would interpret self‐perceived improvement or the lack thereof after 2 weeks of tDCS in terms of confidence of treatment assignment. A quarter of individuals equated a decrease in ataxia severity with definite allocation to real tDCS, while another 60% thought that this would make randomization to real tDCS more likely than sham tDCS (Fig. 2C). Only 2 patients acknowledged that subjectively perceived improvement might equally well indicate allocation to the sham group. Conversely, 65% ascribed the absence of short‐term improvement to either definite or probable assignment to sham tDCS (Fig. 2D).

Beforehand, only 4 out of 20 patients with SCA3 did not have a preference to which group they would be randomized. However, regardless of treatment allocation, 60% of respondents indicated that they were willing to participate again if we would redo the study. The most commonly reported reasons to participate were an opportunity to help other people with SCA3 (especially the next generation, 30%), only help themselves (20%), help other patients and themselves (30%), advance science (15%), or question left unanswered (5%).

To ascertain whether the aforementioned findings are treatment specific, the final questions contained a hypothetical scenario with an oral drug and an identical study aim. After 3 months of daily intake, 80% of individuals thought that they would have reduced ataxia severity if randomized to the real drug (Fig. 1E). Notably, an average improvement of 40.5% (SD, 27.1%) was expected (Fig. 2B), which was not significantly different from the anticipated change after tDCS (*P* = 0.16). When the drug was discontinued after 3 months, 55% believed that they would still have less severe ataxia at 1‐year follow‐up compared with baseline (Fig. 1F).

There were no correlations between the expected degree of improvement after 2 weeks of tDCS and age, SARA score, disease duration, CCAS‐S score, educational level, and percent change in SARA score (Pearson's *r* range: − 0.23 to 0.14; all *P* values > 0.3).

## 3 Discussion

This study shows that the vast majority of patients with SCA3 in a randomized, double‐blind, placebo‐controlled trial expected short‐term and long‐term health benefits when allocated to the active treatment arm in spite of an informed consent process with seemingly well‐balanced information. Most participants associated a lack of symptomatic improvement with probable or definite assignment to the sham group. High expectations of benefit were neither related to the type of intervention—device‐based or a (hypothetical) oral drug—nor to clinical or demographic characteristics.

Trials in patients with heredodegenerative ataxias have thus far examined the potential of interventions to provide symptomatic improvement for a period of weeks to months. Only a small fraction of studies had a follow‐up duration of 1 year or longer. Strikingly, the recently published ATRIL trial investigating the efficacy of riluzole in spinocerebellar ataxia type 2 (SCA2), as well as our own tDCS trial, found absolute changes in SARA score at 1‐year follow‐up in the placebo arms (ATRIL: median 0.3 points; SCA3‐tDCS: mean −0.2 points) that were considerably different from the mean annual increases in natural history studies of patients with SCA2 and SCA3 (1.49 and 1.56 points, respectively).^3^, ^5^, ^8^ These findings suggest that the mere act of participating in a therapeutic trial induces a distinct clinical progression pattern—at the level of rating scale scores—in patients with degenerative ataxias, which might be attributed to expectations of benefit but also to improved care and more attention from physicians (ie, the Hawthorne effect).^7^

Although all patients probably hoped for the best personal outcome (ie, therapeutic optimism), which is usually not an ethical concern, we explicitly asked them about their expectations and not about hopes. Besides the common overestimation of direct benefits (ie, therapeutic misestimation), an important observation of this study was that one‐fifth of respondents stated that they had participated only to reduce the severity of their own symptoms. This response is at odds with the principal purpose of a clinical trial, namely, to produce generalizable knowledge by answering a scientific question that might eventually benefit future patients, and implies that these individuals failed to appreciate the difference between research and regular clinical care (ie, therapeutic misconception).^9^ Misunderstanding the nature of research is ethically problematic as it impacts a patient's decision to participate and therefore undermines the informed consent process.^10^ Of note, misconceptions do not exclusively affect patients but have also been described in physicians recruiting patients for trials.^11^

In the field of movement disorders, therapeutic misestimation and therapeutic misconception are not confined to individuals with ataxia. A recent study showed that more than half of patients with Parkinson's disease expected long‐term improvement if randomized to the active treatment arm in a disease‐modification trial.^12^ Worse cognitive performance, older age, and impaired mobility were associated with higher expectations. Furthermore, therapeutic misconception occurred significantly more often in patients with Huntington's disease than either primary caregivers or asymptomatic mutation carriers.^13^

Designing clinical trials in an area where hopes and expectations of participants and their families may have an unmeasurable influence on outcomes entails considerable difficulties. Preventing or reducing therapeutic misestimation and therapeutic misconception represents an important challenge for interventional studies in neurodegenerative disorders. Although evidence‐based guidelines to accomplish this task are lacking, we reflect on some potential strategies in the Supplementary Materials.

This study has several limitations. In addition to the restricted sample size, questionnaires were completed during trial conduct rather than at enrollment. Importantly, however, disclosure of treatment allocation had not yet occurred and there were no differences in anticipated improvement following real tDCS between patients in both study arms. Moreover, the decision whether or not to participate in a trial may have been prompted (mistakenly) by a priori expectations, which could have increased the prevalence of therapeutic misestimation in our study population. Finally, there was relatively little variability in delta SARA score after 2 weeks of tDCS, which, together with the restricted sample size, might have contributed to the lack of association between expected short‐term improvement and actual outcome.

Still, we believe that our findings have important implications for the design of future therapeutic trials. First, high expectations of benefit feed the placebo response, may partly explain the considerable proportion of placebo responders in previous ataxia trials,^14^ and might render outcomes of open‐label studies in patients with ataxia potentially unreliable. Second, discrepancies in long‐term progression rates between trial and real‐world data once again underscore the necessity of using a placebo arm to draw sound conclusions, especially about the efficacy of future disease‐modifying therapies. Third, given the high prevalence of therapeutic misestimation and therapeutic misconception, specific care should be taken to properly inform and educate patients with ataxia about the aims of a clinical trial prior to participation. Finally, we encourage interventional studies in degenerative ataxias—and movement disorders in general—to (1) implement a questionnaire at baseline like the one used here to inventory expectations of benefit beforehand and (2) evaluate possible changes in perceived treatment during the course of a trial.^15^

## Author Roles

(1) Research Project: A. Conception, B. Organization, C. Execution; (2) Statistical Analysis: A. Design, B. Execution; (3) Manuscript Preparation: A. Writing of the First Draft, B. Review and Critique.

R.P.P.W.M.M.: 1A, 1B, 1C, 2A, 2B, 3A

B.P.C.v.d.W.: 3B

## Financial Disclosures

R.P.P.W.M.M. receives research support from the National Ataxia Foundation. B.P.C.v.d.W. receives research support from ZonMw, Hersenstichting, Gossweiler Foundation, and Radboud University Medical Center. He has served on advisory boards for uniQure and Servier and receives royalties from Bohn Stafleu van Loghum–Springer Nature.

## Supporting information


**Appendix S1.** Supporting Information

## Data Availability

Anonymized data will be shared by the corresponding author upon reasonable request from a qualified investigator.
