# Supplementary material for: Therapeutic Misestimation in Patients with Degenerative Ataxia: Lessons from a Randomized Controlled Trial
Source: Mov Disord. 2022 Oct 19;38(1):133–7. doi: 10.1002/mds.29252 (PMC10092424; doi:10.1002/mds.29252)
Supplement: Supplementary file 1 — Appendix S1. Supporting Information [file MDS-38-133-s001.docx]

**Supplementary data**

**A – Questionnaire**

**B – Possible strategies to reduce or prevent therapeutic misestimation and therapeutic misconception**

**C – Supplementary figure**

**A – Questionnaire**

The questions below refer to the period **just before you participated in the cerebellar tDCS trial**. There are no trick questions and no wrong answers. Go back to the expectations you had at that time, be honest when answering the questions, and complete the questionnaire preferably by yourself without consulting others. Always pick only one answer. Thank you for your time!

**Question 1.**

If I should receive real tDCS daily for 2 consecutive weeks, I expect that after 2 weeks I will have:

1. Much fewer coordination deficits compared to the period just before participating in the trial.
2. Somewhat fewer coordination deficits compared to the period just before participating in the trial.
3. Unchanged coordination deficits compared to the period just before participating in the trial.
4. Somewhat more coordination deficits compared to the period just before participating in the trial.
5. Much more coordination deficits compared to the period just before participating in the trial.

**Question 2.**

If I should receive real tDCS daily for 2 consecutive weeks, I expect that after 1 year I will have:

1. Much fewer coordination deficits compared to the period just before participating in the trial.
2. Somewhat fewer coordination deficits compared to the period just before participating in the trial.
3. Unchanged coordination deficits compared to the period just before participating in the trial.
4. Somewhat more coordination deficits compared to the period just before participating in the trial.
5. Much more coordination deficits compared to the period just before participating in the trial.

**Question 3.**

If I should receive placebo tDCS daily for 2 consecutive weeks, I expect that after 2 weeks I will have:

1. Much fewer coordination deficits compared to the period just before participating in the trial.
2. Somewhat fewer coordination deficits compared to the period just before participating in the trial.
3. Unchanged coordination deficits compared to the period just before participating in the trial.
4. Somewhat more coordination deficits compared to the period just before participating in the trial.
5. Much more coordination deficits compared to the period just before participating in the trial.

**Question 4.**

If I should receive placebo tDCS daily for 2 consecutive weeks, I expect that after 1 year I will have:

1. Much fewer coordination deficits compared to the period just before participating in the trial.
2. Somewhat fewer coordination deficits compared to the period just before participating in the trial.
3. Unchanged coordination deficits compared to the period just before participating in the trial.
4. Somewhat more coordination deficits compared to the period just before participating in the trial.
5. Much more coordination deficits compared to the period just before participating in the trial.

**Question 5.**

If my coordination deficits improve after 2 consecutive weeks of daily tDCS, this would indicate that:

1. I received real tDCS.
2. I more likely received real tDCS rather than placebo tDCS.
3. I could have received real tDCS as likely as placebo tDCS.
4. I more likely received placebo tDCS rather than real tDCS.
5. I received placebo tDCS.

**Question 6.**

If my coordination deficits do *not* improve after 2 consecutive weeks of daily tDCS, this would indicate that:

1. I received real tDCS.
2. I more likely received real tDCS rather than placebo tDCS.
3. I could have received real tDCS as likely as placebo tDCS.
4. I more likely received placebo tDCS rather than real tDCS.
5. I received placebo tDCS.

**Question 7.**

Before participating in this study:

1. I had a preference to be randomized to the real tDCS group.
2. I had a preference to be randomized to the placebo tDCS group.
3. I did not have a preference to which group I would be randomized.

**Question 8.**

If I should receive real tDCS daily for 2 consecutive weeks, I expected – before participating in this study – that my coordination deficits after those 2 weeks would improve by:

…% (fill out a number between 0 and 100).

**Question 9.**

This is a hypothetical question. Suppose that we would conduct this study again in a similar way. Would you participate again?

1. Yes, regardless if I would be allocated to receive real tDCS or placebo tDCS.
2. Yes, but only if I would be allocated to receive real tDCS.
3. Yes, but only if I would be allocated to receive placebo tDCS.
4. No, I would not participate a second time.

**Question 10.**

What was the most important reason for you to participate in this study?

…………………………

**Question 11.**

This is a hypothetical question/situation. Suppose that we had not conducted a clinical trial with daily tDCS for 2 consecutive weeks, but instead we conducted a trial with an oral drug that should be taken daily for a period of 3 months (of course extensively tested before for safety). The aim of the study would be identical to that of the tDCS trial. Similarly, one group would receive the real drug, while the other would receive a placebo drug.

If I should take the real drug daily for 3 consecutive months, I expect that after 3 months I will have:

1. Much fewer coordination deficits compared to the period just before participating in the trial.
2. Somewhat fewer coordination deficits compared to the period just before participating in the trial.
3. Unchanged coordination deficits compared to the period just before participating in the trial.
4. Somewhat more coordination deficits compared to the period just before participating in the trial.
5. Much more coordination deficits compared to the period just before participating in the trial.

**Question 12.**

If I should take the real drug from the previous question daily for 3 consecutive months, I expect that after 1 year I will have:

1. Much fewer coordination deficits compared to the period just before participating in the trial.
2. Somewhat fewer coordination deficits compared to the period just before participating in the trial.
3. Unchanged coordination deficits compared to the period just before participating in the trial.
4. Somewhat more coordination deficits compared to the period just before participating in the trial.
5. Much more coordination deficits compared to the period just before participating in the trial.

**Question 13.**

If I should receive the real drug from the previous questions daily for 3 consecutive months, I expect that my coordination deficits after those 3 months would improve with:

…% (fill out a number between 0 and 100).

**B – Possible strategies to reduce or prevent therapeutic misestimation and therapeutic misconception**

Designing clinical trials in an area where hopes and expectations of participants and their families may have an unmeasurable influence on outcomes entails considerable difficulties. Preventing or reducing therapeutic misestimation and therapeutic misconception represents an important challenge for interventional studies in neurodegenerative disorders. Although evidence-based guidelines to accomplish this task are lacking, key elements probably include timely recognition of the phenomenon by investigators and clinicians in the field and proper education and preparation of patients well before their actual enrolment. As disease-modifying therapies will perhaps become available for the most common types of dominantly inherited ataxia and the first phase I studies assessing safety and tolerability are already on the horizon, we propose that the time is now to start addressing these issues. Not only trial investigators but also patient organisations should play a role in setting realistic expectations by arranging physical meetings and interactive webinars for its members in which the general purpose and procedure of randomized controlled studies are explained as well as the fundamental differences with routine (individualized) clinical care. Indeed, scientific reframing through an educational intervention has proven effective in reducing therapeutic misconception in hypothetical clinical trials (Christopher et al. *PLoS One* 2017). Involvement of a (neutral) research ethicist in the consent process has been suggested as an additional means to properly balance optimism with realism (McCormick, *AMA J Ethics* 2018). Regarding the written and verbal information provided to patients, a careful choice of words is imperative (e.g., preferably “study compound” rather than “drug” or “treatment”) (De Bot, *J Huntingtons Dis.* 2019). Although it may seem obvious from a physician’s perspective, investigators conducting trials with disease-modifying therapies should emphasize that any function that is already lost will not return. Furthermore, limitations of previous successful cell and animal studies may be mentioned, if appropriate, given the considerable differences between these models and humans (Aartsma-Rus, *Neuromuscul Disord.* 2011). Lastly, we recommend to explicitly discuss the possibility of neutral outcomes at enrolment, continue ascertaining consent during the trial, and correct misconceptions as they occur.

**C – Supplementary figure**

**Baseline assessment**

**2-week follow-up**

**1-year**

**follow-up**

**November 2018 – April 2019**

**November 2019 – April 2020**

**Expectations**

**questionnaire**

**May 2021**

**Disclosure of treatment**

**assignment**

**August 2021**

**Supplementary Figure 1.** Timeline representing the most important events in the SCA3-tDCS study. Twenty patients with spinocerebellar ataxia type 3 (SCA3) were randomly assigned to receive ten sessions of cerebellar anodal transcranial direct current stimulation (tDCS) or sham tDCS within an intervention period of two weeks (i.e., five days per week for two consecutive weeks with a two-day weekend break). Short-term effects were assessed directly after the last tDCS session, while long-term effects were evaluated after three months, six months, and twelve months. For the sake of simplicity and in line with the two time points included in the questionnaire, the three-month and six-month follow-up visits have not been included in the figure. The expectations patients had just before participation in this study were explored retrospectively, but well before disclosure of treatment arm assignment. At the time of completion of the questionnaire, patients were thus unaware of their randomization status. Because of the COVID-19 pandemic, one-year follow-up visits of the last participants (planned for March/April 2020) had to be cancelled. These were caught up in person several months later, which explains the time interval to disclosure of treatment arm assignment.
